# Significantly Different Lipid Profile Analysis of *Litopenaeus vannamei* under Low-Temperature Storage by UPLC-Q-Exactive Orbitrap/MS

**DOI:** 10.3390/foods10112624

**Published:** 2021-10-29

**Authors:** Shengnan Wang, Yongshi Chen, Yu Chen, Peng Liang, Jie Pang, Beiwei Zhu, Xiuping Dong

**Affiliations:** 1 College of Food Science, Fujian Agriculture and Forestry University, Fuzhou 350002, China; 1180940006@fafu.com (S.W.); 3190910003@fafu.edu.cn (Y.C.); 3190910004@fafu.edu.cn (Y.C.); liangpeng0412@fafu.edu.cn (P.L.); 2 National Engineering Research Center of Seafood, Collaborative Innovation Center of Seafood Deep Processing, School of Food Science and Technology, Dalian Polytechnic University, Dalian 116034, China; zhubw@dlpu.edu.cn

**Keywords:** lipidomics, *Litopenaeus vannamei*, low-temperature storage, lipid classes, UPLC-Q-Exactive Orbitrap/MS

## Abstract

Low-temperature storage is one of the most important preservation methods for aquatic product storage. However, the effects of low-temperature storage on the lipid profiles of shrimp are unclear. Herein, UPLC-Q-Exactive Orbitrap/MS combined with LipidSearch software was applied to analyze the effect of three low storage temperatures (4 °C, −2 °C, and −18 °C) on the lipidomics of *Litopenaeus vannamei*. A total of 15 lipid classes were analyzed, and PC, PE, DG, and TG accounted for vast majority of peak areas. Furthermore, 531 individual lipid variables enriched in 12 metabolic pathways were identified via bioinformatics analysis methods. A total of 56 significantly different lipid molecular species (55 belonging to PC, PE, DG, and TG) were selected as potential biomarkers of lipid oxidation via correlational analysis between physical properties (texture and color) and individual lipid variables. The results indicated that the three low storage temperatures caused different effects on the lipidomics profile of *L. vannamei*, and PC, PE, DG, and TG could become potential focuses in further studies of lipid oxidation in *L. vannamei*.

## 1. Introduction

Shrimp (*Litopenaeus vannamei*) is one of the widely consumed forms of seafood, owing to its enormous nutritional and economic value, including high levels of protein and functional lipids, and low levels of cholesterol [1,2,3]; it also contains abundant long-chain polyunsaturated fatty acids (PUFAs)—especially eicosapentaenoic acid (EPA) and docosahexaenoic acid (DHA). However, despite these excellent characteristics, its quality deteriorates very easily after dead. Thus, it must be preserved soon after harvest in order to prolong its shelf life. Low-temperature storage methods (e.g., refrigerated storage (0–4 °C), particle-freezing storage (−1–−4 °C), and frozen storage (−18–−4 °C) are well known to be used as essential preservation methods in the aquatic products supply chain [4,5,6].

Despite this approach being known to prolong shelf life, many biochemical reactions still occur in seafood during storage. Lipid oxidation is an inevitable process of deterioration, resulting in considerable degradation of various qualities, such as flavor, texture, color, and nutritive value [7]. Previous studies have shown that UFAs degrade into aldehydes, ketones, and lower fatty acids during the lipid oxidation process [8,9]. ω-3 PUFAs are susceptible to peroxidation because of free radicals, resulting in an unpleasant odor and potentially toxic biological effects [10]. Lipid oxidation was evaluated by measuring hydroperoxides and conjugated dienes, revealing the mechanism of producing thiobarbituric acid (TBA) oxidation products [11,12]. However, specific lipid profile information after low-temperature storage has rarely been reported until now.

To provide comprehensive lipidomics profile information, UPLC-Q-Exactive Orbitrap/MS has been developed and utilized in the quantitative tracing of lipid components of foods. In general, successful applications in foods are mainly classified into three aspects: lipid composition characterization, adulteration, and food traceability [13,14,15]. Shi et al. identified eight lipid species variables (including 137 individual lipid variables), demonstrating via UPLC-Q-Exactive Orbitrap/MS that different thermal processing methods have different effects on the lipid profile of tilapia fillets [16]. Zhang et al. reported 22 significantly different lipids between fresh and stored rice via lipidomics analysis [17]. However, to the best of our knowledge, the lipid analysis method has not been applied to analyze the effects of cold storage on *L. vannamei* and potential biomarkers of lipid oxidation via correlational analysis with quality parameters.

Therefore, the object of this work is to investigate the lipidomics profile information of *L. vannamei* between fresh and cold storage under three low-temperature storage methods (4 °C, −2 °C, and −18 °C), and to identify the potential biomarkers of lipid oxidation in *L. vannamei* via correlational analysis between physical properties (texture and color) and significantly different lipids under the three low-temperature storage methods.

## 2. Materials and Methods

### 2.1. Shrimp Preparation and Reagents

Shrimps (*L. vannamei*) with a height of ~15 cm were purchased from Yonghui supermarket (Fuzhou, China) and transported with ice for 1 h. Upon arriving, all 24 samples were washed under flowing deionized water and packed into 4 bags (6 samples for each bag). Of these 4 bags, 3 were randomly kept under three temperature conditions- 4 °C (refrigerated treatment, RT), −2 °C (particle-freezing treatment, PFT), and −18 °C (frozen treatment, FT) for low-temperature storage (for 10, 30, and 60 days, respectively), while one was treated as a fresh group (RAW) for further analysis.

Ammonium formate was purchased from Sigma-Aldrich (Shanghai, China). Chloroform methanol, acetonitrile, isopropanol, and acetonitrile were purchased from Thermo Fisher Scientific (Waltham, MA, USA). All other common reagents for lipid extraction were purchased from Tedia Company Inc. (Fairfield, OH, USA).

### 2.2. Physical Properties Analysis

Texture profile analysis (TPA) and color analysis (L*, a*, and b* values) were measured as the physical properties to be analyzed in this work. The TPA of the second segment in shrimp was measured with a texture analyzer (Stable Micro System, Haste Hill, England) using a circular probe (P/50) with the following modes: two constant cycles, an interrupted speed of 1.0 mm/s, and 50% compression. Then, the color of the minced shrimp samples was tested with a colorimeter (angle 10°, illuminant D65) (ADCI-60-C, Beijing, China). This colorimeter was standardized using a standard black and white blank. The denotation of each parameter (L*, a*, and b*) of color was lightness (0 = black, 100 = white), redness/greenness (+*a* = red, −*a* = green), and yellowness/blueness (+*b* = yellow, −*b* = blue), respectively. Shrimp in the FT group were examined after thawing for 0.5 h at room temperature. All results were determined from 6 samples.

### 2.3. Lipid Extraction

The lipid extraction followed the Folch and Bligh method, with slight modifications [18,19]. In this method, 100 mg of minced shrimp was mixed with 750 μL of chloroform–methanol solution (2:1 ratio), ground using a grinding apparatus (Scienta-48, Ningbo, China) for 1.5 min at 60 Hz, and then vortexed (QL-866 Vortex Mixer, Kylin-Bell, Ningbo, China) for 30 s after adding 190 μL of double-distilled water (Millipore, MA, USA). The mixture was centrifuged (H1850-R, Hunan, China) for 5 min at 12,000 rpm to obtain the lower layer fluid. The fluid was extracted once following the steps described above, and then dried in a vacuum. The residue was filtered (0.22 μm) after re-dissolving in 200 μL of isopropanol solution for subsequent MS analysis.

### 2.4. Instrument Conditions

The lipid profile of *L. vannamei* during storage was determined by applying UPLC-Q-Exaction Orbitrap/MS (Thermo Fisher Science, CA, USA) combined with a heated electrospray ionization probe. The lipid extracts of *L. vannamei* were separated using an ACQUITY UPLC BEH C18 column (2.1 × 100 mm, 1.7 μm, Waters, MA, USA).

A binary solvent system was applied for eluting lipid samples [20]. Mobile phase A: acetonitrile: water (60:40 ratio), including 0.1% formic acid and 10 mM ammonium formate. Mobile phase B: isopropanol: acetonitrile (90:10 ratio), including 0.1% formic acid and 10 mM ammonium formate. A 28-min gradient elution with a flow speed of 0.25 mL/min was performed as follows: initial A equilibrium for 5 min, 50% B to 100% B for 21 min, followed by 100% B for 10 min, and re-equilibration with 50% B for 8 min.

Data were acquired in mass range of *m*/*z* 150–2000 in positive and negative ionization modes with dependent MS/MS acquisition; a resolution of 35,000 was chosen for full-scan and fragment spectra, respectively. The device conditions were set as follows: spray voltages of 3.5 kV (ESI^+^) and 2.5 kV (ESI^−^), capillary temperature of 325 °C, sheath gas flow speed of 30 Arb, and auxiliary gas flow speed of 10 Arb.

### 2.5. Statistics

Peak areas of lipid molecular species were analyzed using LipidSearch 4.0 software (Thermo Fisher Scientific, CA, USA). Heatmap imaging, partial least squares discriminant analysis (PLS-DA), and variable importance in projection (VIP) analysis were performed in MetaboAnalyst 5.0 (https://www.metaboanalyst.ca accessed on 10 September 2021) [20]. The mean values and standard deviations were calculated using Microsoft Excel software. One-way ANOVA was carried out using SPSS software (version 25.0) to test the significant differences (*p* < 0.05) of physical properties and lipid levels during low-temperature storage, and highly significant differences (*p* < 0.01) between physical properties and lipid species. In addition, Pearson’s correlation analysis of individual lipid variables and physical quality characteristics (including L*, a*, b*, springiness, gumminess, cohesiveness, chewiness, and hardness) of *L. vannamei* muscles was carried out using SPSS software. All results were determined from 6 samples.

## 3. Results and Discussion

### 3.1. Quality Analysis of Texture and Color

Texture and color are considered to be crucial sensory parameters to consumers, who will make negative decisions if the shrimp quality (freshness) does not match their expectations [21]. The results of physical properties (L*, a*, b*, springiness, cohesiveness, gumminess, and hardness) are shown in Table 1. It is clear that the color parameters (L*, a*, and b* values) were significantly changed after low-temperature storage (*p* < 0.05). For parameters a* and b*, the four storage conditions showed the following order: RT > PFT > FT > RAW. This was because of a biochemical mechanism called polyphenol oxidase oxidation, which oxidizes phenols to quinones, following which polymerization takes place, leading to melanosis [22,23]. Therefore, the color surface that was initially translucent becomes increasingly opaque, and black spots are formed. Finally, the body of the shrimp turns red.

Moreover, values of texture parameters (springiness, cohesiveness, gumminess, and hardness) in four conditions displayed the following order: RAW > FT > PFT > RT; this may be due to differences caused by the inherent variation of shrimp (i.e., the degradation of myofibrillar proteins caused by microbial decomposition and autolysis, leading to mushiness of shrimp) under different low-temperature storage conditions [24]. Compared with the RT and PFT groups, the FT group had no significant decrease (*p* > 0.05) in cohesiveness, gumminess, or hardness, indicating that textural properties are not easily influenced by muscle softening and gap formation under lower temperatures [25].

### 3.2. Lipid Species Differences

**Figure 1:**
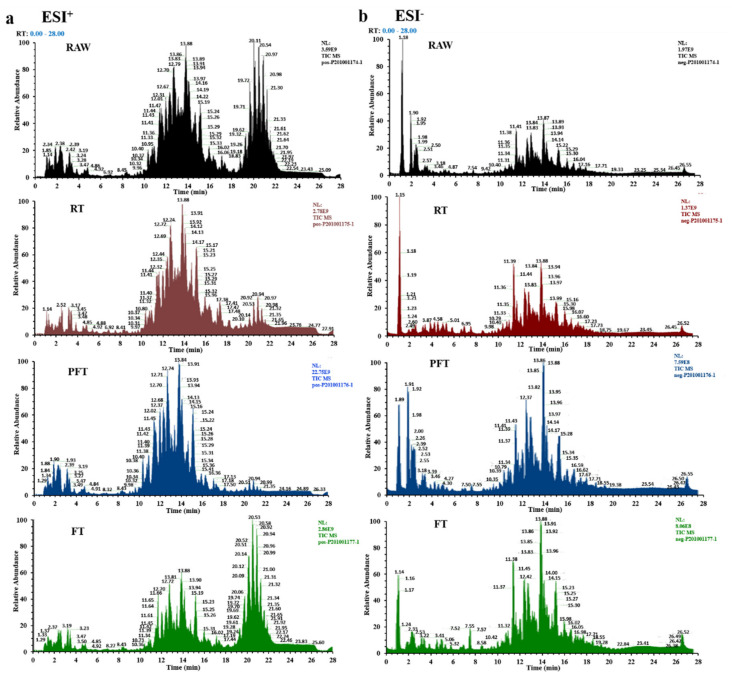
UPLC-Exactive Orbitrap/MS base peak intensity chromatograms of *L. vannamei* acquired in ESI^+^ (**a**) and ESI^−^ (**b**) modes. Note: ESI^+^: positive electrospray ionization; ESI^−^: negative electrospray ionization; RAW: fresh group; RT: refrigerated treatment (4 °C); PFT: particle-freezing treatment (−2 °C); FT: frozen treatment (−18 °C).

Figure 1 presents the mass spectra of lipidomics profiles between fresh and cold-treated shrimp samples at three different temperatures. In ESI^+^ mode (Figure 1a), most of the signals are attributed to Cer, DG, LPC, LPE, LPS, PC, PE, SM, and TG, while Cer, HexCer, LPC, LPE, LPG, LPI, PC, PE, PG, PI, and PS are distributed in ESI^−^ mode (Figure 1b). The lipid molecular species under different low-temperature storage conditions are shown in Table S1. The total number of lipid molecular species is summarized in Table 2. Based on the number of lipid molecular species, *L. vannamei* is mainly composed of PC, PE, Cer, SM, DG, and TG, whereas, PI, PG, PS, LPC, LPE, LPI, LPG, LPS, and HexCer are also found in small concentrations. The total number of lipid molecular species in the three low-temperature storage conditions showed no significant decrease compared with fresh shrimp. Although the abundance of PC, PI, and LPE in the RT group and PG and PS in the PFT group decreased compared with other groups, the abundance of LPI and TG in the RT and PFT groups decreased. Compared with the RT and PFT groups, the number of lipid molecular species in the FT group decreased slightly. It is worth noting that the abundance of PE decreased, and LPG and Cer increased, under all three low-temperature storage conditions compared with RAW. The number of lipid molecular species of *L. vannamei* under different low-temperature storage conditions demonstrates that different low-temperature storage conditions have different effects on the number of lipid molecular species, but further analysis is needed.

**Figure 2:**
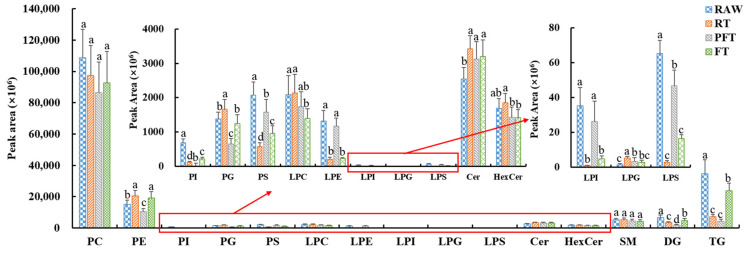
Analysis of peak areas of lipid species in *L. vannamei* after low-temperature storage (values with different letters are significantly different (*p* < 0.05)). Note: RAW: fresh group; RT: refrigerated treatment (4 °C); PFT: particle-freezing treatment (−2 °C); FT: frozen treatment (−18 °C); PC: phosphatidylcholine; PE: phosphatidylethanolamine; PI: phosphatidylinositol; PG: phosphatidylglycerol; PS: phosphatidylserine; LPC: lysophosphatidylcholine; LPE: lysophosphatidylethanolamine; LPI: lysophosphatidylinositol; LPG: lysophosphatidylglycerol; LPS: lysophosphatidylserine; Cer: ceramide; HexCer: hexosylceramide; SM: sphingomyelin; DG: diglyceride; TG: triglyceride.

The lipid categories were displayed and compared using peak areas summarizing the individual lipids in the same class, as shown in Figure 2. The RT group showed de-crease of PC, PI, PS, LPE, LPI, LPS, DG, and TG, and increase of PE, PG, LPG, Cer, and HexCer, with no significant differences in LPC or SM. Unlike the RT group, the PFT group showed decrease of PE, PG, LPG, and HexCer, with an obvious difference in the numbers of lipid species except for PC, LPE, LPI, and SM. Unlike the PFT group, the FT group showed increase of PE, and obvious differences in LPE, LPI, and HexCer. As compared to the RAW group, the amounts of PI, PS, LPS, DG, and TG were decreased, while that of Cer was increased significantly. This may have great potential in terms of lipid composition changes during low-temperature storage.

### 3.3. Multivariate Statistical Analysis and Lipid Metabolism Pathways

**Figure 3:**
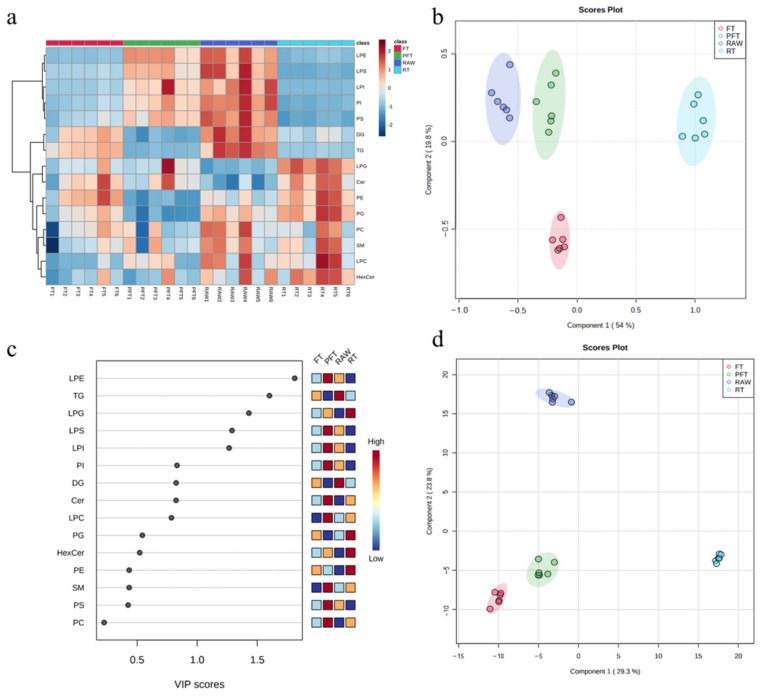
Multivariate statistical analysis of lipids in *L. vannamei* after low-temperature storage. (**a**) Heatmap of lipid species; each colored cell on the map relates to the peak area value of different lipid species; red indicates high, and blue indicates low. (**b**) PLS-DA score plot of lipid species. (**c**) VIP scores of lipid species in PLS-DA; the colored boxes on the right indicate the relative concentrations of different lipid species; red indicates high, and blue indicates low. (**d**) PLS-DA score plot of individual lipids. Note: RAW: fresh group; RT: refrigerated treatment (4 °C); PFT: particle-freezing treatment (−2 °C); FT: frozen treatment (−18 °C); PC: phosphatidylcholine; PE: phosphatidylethanolamine; PI: phosphatidylinositol; PG: phosphatidylglycerol; PS: phosphatidylserine; LPC: lysophosphatidylcholine; LPE: lysophosphatidylethanolamine; LPI: lysophosphatidylinositol; LPG: lysophosphatidylglycerol; LPS: lysophosphatidylserine; Cer: ceramide; HexCer: hexosylceramide; SM: sphingomyelin; DG: diglyceride; TG: triglyceride.

To further understand the variable distributions, multivariate statistical analysis was conducted to explore the differences in shrimp samples under different low-temperature storage conditions, and to figure out the candidate markers for assessing lipid oxidation of cold-treated shrimp. As shown in Figure 3a, a heatmap was used to investigate the intuitive distribution of peak areas of lipid species in the fresh and cold-treated groups, so as to further confirm the analysis in Figure 2. It could be observed that, compared with the RAW group, peak area of LPE, LPS, LPI, PI, PS, DG, and TG decreased under the low-temperature storage conditions, while that of LPG and Cer increased. This demonstrates that low-temperature storage alter the content of lipid species of shrimp to a large extent. We also observed some different colors among the six repeats for each group, owing to the individual disparity of *L. vannamei*.

PLS-DA—a multiple linear regression method for finding the direction of maximum covariance between the dataset (X) and class membership (Y)—was applied to study the changes in lipid species of *L. vannamei* after different low-temperature storage conditions [26]. As shown in the score plot in Figure 3b, the RAW, RT, PFT, and FT groups were classified based on the first two principal components, and the cumulative contribution rate was 73.8%. Additionally, the estimated VIP scores of lipid species of different cold-treated *L. vannamei* are shown in Figure 3c. Five lipid species (LPE, TG, LPG, LPS, and LPI) accounted for the most significant contribution (VIP score > 1), which could be the lipid species with the best potential for differentiating the cold-treated *L. vannamei*.

Furthermore, individual lipids of the four kinds of cold-treated shrimp in PLS-DA, along with their VIP scores, were also estimated. As shown in the score plot of individual lipids (Figure 3d), the four conditions (RAW, RT, PFT, and FT) were classified based on the first two principal components, and the cumulative contribution rate was 53.1%. Meanwhile, a total of 531 individual lipid variables (including 62 PCs, 56 PEs, 17 PIs, 9 PGs, 17 PSs, 3 LPCs, 13 LPEs, 5 LPIs, 2 LPGs, 5 LPSs, 14 Cers, 7 HexCers, 5 SMs, 36 DGs, and 280 TGs) accounted for significant contributions based on VIP scores > 1 (as shown in Table S2). These individual lipids could also be used to differentiate the different cold-treated *L. vannamei*. In addition, KEGG (Kyoto Encyclopedia of Genes Genomes) pathway analysis was performed on these 531 lipid molecular species to elucidate the lipid changes of *L. vannamei* in metabolic pathways after low-temperature storage. These lipid molecular species were significantly enriched in KEGG pathways including glycosylphosphatidylinositol (GPI)-anchor biosynthesis, glycerophospholipid metabolism, cholesterol metabolism, fat digestion and absorption, regulation of lipolysis in adipocytes, insulin resistance, thermogenesis, linoleic acid metabolism, glycerolipid metabolism, vitamin digestion and absorption, α-linolenic acid metabolism, and arachidonic acid metabolism. Among these pathways, glycosylphosphatidylinositol (GPI)-anchor biosynthesis was the pathway with the most significant difference in lipid involvement, followed by glycerophospholipid metabolism and cholesterol metabolism (as shown in Figure S1).

Glycosylphosphatidylinositol (GPI) anchors as lipid anchors are usually conjunct to the surface of macromolecules and plasma membranes [27]. Many membranous enzymes, receptors, differentiation antigens, and other biologically active proteins are bound to the plasma membrane by GPI [28]. In this work, PE (18:2/20:4) was found to be related to GPI-anchor biosynthesis. The glycerophospholipid metabolism is mainly catalyzed by phospholipases and lysolecithin, and unsaturated fatty acids are metabolic products [29,30]. The contents of PC and PE in shrimp after low-temperature storage were lower than those in the fresh group—such as PC (18:1/14:0), PC (18:3/18:3), PC (44:4), PC (46:0), PC (18:0/16:1), PC (18:4/18:3), PC (16:1/22:5), PC (18:3/20:4), PC (20:3/20:5), and PE (18:2/20:4)—showing that the lipid transformations of PC and PE progressed based on glycerophospholipid metabolism. Similarly to our study, Zhou et al. also reported that the content of PC and PE decreases during low-temperature storage at 4 °C in the mussel *Mytilus edulis* [31]. Moreover, PC and PE hydrolyze during rice storage, as reported by Zhang et al. [17]. We believe that KEGG pathway analysis of significantly different lipid molecular species promotes our understanding of the changes and functions of shrimp lipids after low-temperature storage.

### 3.4. Correlation Analysis

Pearson’s correlational analysis between the 531 individual lipid variables and physical quality characteristics (including L*, a*, b*, springiness, gumminess, cohesiveness, chewiness, and hardness) of *L. vannamei* under low-temperature storage (RT, PFT, and FT compared with RAW) is presented in Table S3. To find further highly significant correlations (*p* < 0.01) with physical parameters, 56 lipid molecular species (including 4 PCs, 3 PEs, 2 PGs, 1 LPG, 4 DGs, and 42 TGs) were selected based on two requirements: R^2^ > |0.9|, and the number of significant correlations with physical parameters for each lipid being no less than two. It should be noted that the total number of lipid molecular species in PC, PE, DG, and TG was 53, constituting a large proportion of all observed molecular species (56), which is consistent with the lipid molecular numbers and peak areas in Section 3.2. As shown in Table 3, PC (11:0/16:1), PC (26:5e), PC (29:2), PC (47:1), PE (15:0/16:1), PE (16:1/14:1), PE (36:3e), PG (16:1/14:0), PG (17:1/16:1), LPG (16:1), DG (16:0/23:0), and DG (18:2e) had highly significant positive correlations with b*, and negative correlations with springiness. DG (36:0e) and DG (38:3e) had highly significant positive correlations with L* and hardness. It is worth mentioning that 41 of the 42 TGs (except for TG (50:6e)) had highly significant correlations with L* and hardness. TG (50:6e) had a highly significant positive correlation with b* and springiness, and a negative correlation with cohesiveness and hardness. Five lipids (including PE (16:1/14:1), TG (14:0/14:0/20:3), TG (18:1/18:2/22:1), TG (22:1/18:2/23:0), and TG (50:6e)) had highly significant correlations with more than two physical parameters. These lipids could be potential biomarkers for quality changes in *L. vannamei*.

## 4. Conclusions

In summary, the lipidomics profiles in fresh (RAW) and cold-treated (RT, PFT, and FT) *L. vannamei* were determined using UPLC-Q-Exactive Orbitrap/MS and lipidomics software. A total of 15 lipid classes were analyzed among the 4 groups of shrimp; among them, PC, PE, DG, and TG accounted for the vast majority of peak areas. A total of 531 individual lipid variables involved in 12 metabolic pathways were identified via bioinformatics analysis methods. In addition, 56 lipid molecular species (55 belonging to PC, PE, DG, and TG) were selected as potential biomarkers of lipid oxidation in *L. vannamei* via correlational analysis between physical properties (texture and color) and 531 individual lipid variables during low-temperature storage. It is worth mentioning that PC, PE, DG, and TG could become potential focuses in further studies of lipid oxidation in *L. vannamei*. This work could present a practical means to evaluate the effect of low-temperature storage on the lipid oxidation of aquatic products, and provide a basic conception for the understanding of lipidomics.

## Figures and Tables

**Table 1:** Physical quality analysis of *L. vannamei* after low-temperatures storage.

	RAW ^1^	RT ^2^	PFT ^3^	FT ^4^
L* ^5^	41.04 ± 0.89 ^a 8^	28.52 ± 1.43 ^d^	31.46 ± 0.54 ^c^	39.48 ± 0.31 ^b^
a* ^6^	0.2 ± 0.13 ^c^	1.75 ± 0.43 ^a^	1.51 ± 0.22 ^a^	0.9 ± 0.12 ^b^
b* ^7^	0.89 ± 0.25 ^b^	4.11 ± 0.58 ^a^	1.41 ± 0.31 ^b^	1.04 ± 0.36 ^b^
Springiness (N)	0.99 ± 0.01 ^a^	0.62 ± 0.05 ^c^	0.9 ± 0.02 ^b^	0.93 ± 0.02 ^b^
Cohesiveness	0.44 ± 0.02 ^a^	0.3 ± 0.02 ^c^	0.39 ± 0.01 ^b^	0.43 ± 0.01 ^a^
Gumminess (g)	816.53 ± 97.59 ^a^	534.17 ± 34.88 ^c^	615.65 ± 20.39 ^b^	757.2 ± 2.95 ^a^
Chewiness (g·mm)	700.66 ± 24.21 ^a^	421.23 ± 24.56 ^d^	490.18 ± 52.15 ^c^	552.06 ± 56.03 ^b^
Hardness (g)	1887.43 ± 85.81 ^a^	1009.8 ± 93.01 ^c^	1362.43 ± 27.81 ^b^	1778.55 ± 117.51 ^a^

Note: ^1^ RAW: fresh group; ^2^ RT: refrigerated treatment (4 °C); ^3^ PFT: particle-freezing treatment (−2 °C); ^4^ FT: frozen treatment (−18 °C); ^5^ L*: lightness (0 = black, 100 = white); ^6^ a*: redness/greenness (+*a* = red, −*a* = green); ^7^ b*: yellowness/blueness (+*b* = yellow, −*b* = blue); ^8^ mean values ± standard deviations for 6 samples. Values in the same row with different letters are significantly different (*p* < 0.05).

**Table 2:** Comparisons of total lipid molecular numbers of different lipid species of *L. vannamei* after low-temperature storage.

Treatments	MS Spectra Mode	PC	PE	PI	PG	PS	LPC	LPE	LPI	LPG	LPS	Cer	HexCer	SM	DG	TG	Sum
RAW	ESI^+^	349	128	10	3	14	42	22	1	0	6	30	0	94	173	621	1493
	ESI^−^	217	203	35	31	88	16	11	5	4	0	121	60	0	0	0	801
RT	ESI^+^	346	129	8	2	14	42	21	1	0	6	30	0	94	173	615	1481
	ESI^−^	217	198	33	31	88	16	10	4	5	0	123	60	0	0	0	795
PFT	ESI^+^	348	124	10	3	14	42	22	1	0	6	30	0	94	171	615	1480
	ESI^−^	217	197	35	28	87	16	11	3	5	0	123	60	0	0	0	792
FT	ESI^+^	349	129	10	2	14	42	21	1	0	6	29	0	94	173	622	1492
	ESI^−^	217	199	35	31	88	16	11	5	5	0	123	59	0	0	0	799

Note: RAW: fresh group; RT: refrigerated treatment (4 °C); PFT: particle-freezing treatment (−2 °C); FT: frozen treatment (−18 °C); ESI^+^: positive electrospray ionization; ESI^−^: negative electrospray ionization; PC: phosphatidylcholine; PE: phosphatidylethanolamine; PI: phosphatidylinositol; PG: phosphatidylglycerol; PS: phosphatidylserine; LPC: lysophosphatidylcholine; LPE: lysophosphatidylethanolamine; LPI: lysophosphatidylinositol; LPG: lysophosphatidylglycerol; LPS: lysophosphatidylserine; Cer: ceramide; HexCer: hexosylceramide; SM: sphingomyelin; DG: diglyceride; TG: triglyceride.

**Table 3:** Pearson’s correlation between significantly different lipid molecular species (R^2^ > |0.9|, *n* ≥ 2) and quality characteristics of *L. vannamei* after low-temperature treatments.

Molecular Species	Ion (m/z)	L* ^1^	a* ^2^	b* ^3^	Springiness	Gumminess	Cohesiveness	Chewiness	Hardness
**PC** ^4^									
(11:0/16:1)	662.48	−0.676	0.589	0.934	−0.902	−0.644	−0.861	−0.575	−0.741
(26:5e)	626.42	−0.677	0.588	0.930	−0.915	−0.637	−0.880	−0.578	−0.755
(29:2)	688.49	−0.625	0.558	0.918	−0.921	−0.628	−0.854	−0.564	−0.718
(47:1)	942.79	−0.711	0.607	0.930	−0.910	−0.679	−0.885	−0.584	−0.781
**PE** ^5^									
(15:0/16:1)	674.48	−0.695	0.577	0.948	−0.930	−0.657	−0.897	−0.592	−0.780
(16:1/14:1)	658.45	−0.732	0.622	0.962	−0.954	−0.708	−0.910	−0.657	−0.804
(36:3e)	750.54	−0.656	0.610	0.919	−0.914	−0.639	−0.851	−0.630	−0.734
**PG** ^6^									
(16:1/14:0)	691.46	−0.717	0.608	0.948	−0.942	−0.697	−0.697	−0.635	−0.798
(17:1/16:1)	731.49	−0.707	0.584	0.956	−0.948	−0.685	−0.685	−0.623	−0.795
**LPG** ^7^									
(16:1)	481.26	−0.707	0.615	0.924	−0.917	−0.655	−0.898	−0.596	−0.789
**DG** ^8^									
(16:0/23:0)	684.65	−0.698	0.558	0.928	−0.917	−0.668	−0.894	−0.574	−0.800
(18:2e)	355.28	−0.696	0.590	0.939	−0.907	−0.650	−0.878	−0.579	−0.766
(36:0e)	633.58	0.937	−0.861	−0.643	0.703	0.866	0.792	0.849	0.902
(38:3e)	633.58	0.937	−0.861	−0.643	0.703	0.866	0.792	0.849	0.902
**TG** ^9^									
(14:0/14:0/20:3)	801.70	−0.909	0.790	0.936	−0.932	−0.847	−0.847	−0.749	−0.939
(16:0/10:0/16:1)	738.66	0.939	−0.805	−0.734	0.746	0.887	0.887	0.785	0.921
(16:0/17:0/22:6)	910.79	0.943	−0.858	−0.641	0.693	0.877	0.877	0.832	0.903
(16:0/18:1/22:1)	937.82	0.952	−0.820	−0.693	0.735	0.880	0.880	0.791	0.920
(16:0/18:1/24:1)	965.85	0.935	−0.788	−0.660	0.685	0.860	0.860	0.756	0.900
(16:0/19:0/22:6)	938.82	0.941	−0.821	−0.650	0.688	0.865	0.865	0.788	0.902
(16:1/12:0/16:1)	764.68	0.946	−0.827	−0.689	−0.689	0.882	0.821	0.800	0.920
(17:0/18:2/18:3)	884.77	0.931	−0.881	−0.635	−0.635	0.903	0.757	0.854	0.890
(18:1/12:0/14:0)	766.69	0.949	−0.823	−0.691	−0.691	0.887	0.822	0.800	0.915
(18:1/12:3/18:1)	814.69	0.923	−0.845	−0.705	−0.705	0.880	0.826	0.840	0.914
(18:1/18:1/21:1)	949.82	0.942	−0.792	−0.658	−0.658	0.859	0.797	0.755	0.903
(18:1/18:2/22:0)	963.84	0.946	−0.799	−0.674	−0.674	0.864	0.816	0.763	0.909
(18:1/18:2/22:1)	961.82	0.957	−0.831	−0.721	−0.721	0.902	0.847	0.812	0.928
(18:1/20:2/22:5)	976.83	0.942	−0.835	−0.647	−0.647	0.876	0.795	0.806	0.906
(18:1/20:5/23:0)	994.88	0.945	−0.845	−0.646	−0.646	0.867	0.791	0.813	0.904
(18:1/22:1/22:6)	1004.86	0.956	−0.820	−0.699	−0.699	0.876	0.828	0.791	0.928
(18:1/22:6/24:1)	1032.90	0.925	−0.746	−0.658	−0.658	0.827	0.796	0.715	0.903
(18:1/24:1/24:1)	1071.00	0.952	−0.863	−0.657	−0.657	0.875	0.800	0.839	0.918
(19:0/18:1/22:6)	964.83	0.945	−0.822	−0.653	−0.653	0.868	0.798	0.782	0.906
(19:0/22:1/22:1)	1030.97	0.946	−0.872	−0.645	−0.645	0.883	0.790	0.846	0.905
(19:1/18:1/22:6)	962.82	0.949	−0.849	−0.645	−0.645	0.884	0.785	0.813	0.906
(20:1/18:1/22:6)	976.83	0.946	−0.796	−0.686	−0.686	0.872	0.823	0.757	0.910
(20:1/18:1/24:0)	1016.96	0.949	−0.822	−0.664	−0.664	0.875	0.806	0.788	0.912
(20:1e/20:5/20:5)	956.81	0.946	−0.824	−0.675	−0.675	0.895	0.800	0.784	0.911
(22:0/18:2/20:5)	961.82	0.955	−0.841	−0.722	−0.722	0.893	0.845	0.843	0.935
(22:1/18:2/22:6)	1002.85	0.941	−0.876	−0.672	−0.672	0.886	0.814	0.867	0.913
(22:1/18:2/23:0)	1028.96	0.951	−0.900	−0.644	−0.644	0.887	0.789	0.869	0.903
(22:2/18:2/22:6)	1000.83	0.937	−0.850	−0.649	−0.649	0.883	0.790	0.826	0.906
(24:1/18:2/22:6)	1030.88	0.945	−0.800	−0.671	−0.671	0.863	0.803	0.758	0.906
(24:1/18:2/23:0)	1056.99	0.948	−0.857	−0.652	−0.652	0.877	0.794	0.834	0.912
(24:1/18:2/24:1)	1068.99	0.948	−0.863	−0.651	−0.651	0.884	0.794	0.835	0.909
(25:0/18:1/18:1)	1002.94	0.939	−0.868	−0.636	−0.636	0.880	0.781	0.843	0.901
(26:0/18:1/18:2)	1014.94	0.945	−0.820	−0.651	−0.651	0.865	0.799	0.783	0.907
(26:0/18:2/18:2)	1012.93	0.936	−0.835	−0.640	−0.640	0.879	0.774	0.802	0.900
(28:0/18:1/18:1)	1044.99	0.943	−0.831	−0.647	−0.647	0.879	0.798	0.795	0.902
(28:0/18:1/18:2)	1042.97	0.949	−0.841	−0.662	−0.662	0.879	0.803	0.812	0.912
(29:0/18:1/18:1)	1059.00	0.952	−0.835	−0.653	−0.653	0.860	0.800	0.793	0.903
(30:0/18:1/18:1)	1073.02	0.943	−0.817	−0.650	−0.650	0.860	0.796	0.788	0.907
(50:6e)	831.68	−0.867	0.759	0.959	0.959	−0.817	−0.957	−0.734	−0.906
(59:6)	966.85	0.942	−0.823	−0.650	−0.650	0.858	0.800	0.794	0.908
(62:6e)	994.92	0.932	−0.875	−0.624	−0.624	0.903	0.769	0.839	0.886
(63:9)	1016.86	0.949	−0.861	−0.648	−0.648	0.876	0.797	0.826	0.908

Notes: All of the lipid molecular species selected based on the condition (R^2^ > |0.9|, *n* ≥ 2) had highly significant correlations (*p* < 0.01) with physical properties; ^1^ L*: lightness (0 = black, 100 = white); ^2^ a*: redness/greenness (+*a* = red, −*a* = green); ^3^ b*: yellowness/blueness (+*b* = yellow, −*b* = blue); ^4^ PC: phosphatidylcholine; ^5^ PE: phosphatidylethanolamine; ^6^ PG: phosphatidylglycerol; ^7^ LPG: lysophosphatidylglycerol; ^8^ DG: diglyceride; ^9^ TG: triglyceride.

## Data Availability

The data presented in this study are available on request from the corresponding authors.

## Supplementary Materials

The following are available online at https://www.mdpi.com/article/10.3390/foods10112624/s1: Table S1: Information on lipid molecular species in *L. vannamei* after low-temperature storage; Table S2: VIP scores of significantly different lipid molecular species of *L. vannamei* after low-temperature storage; Table S3: Pearson’s correlation between all significantly different lipid molecular species and quality characteristics of *L. vannamei* after low-temperature storage; Figure S1: Metabolomic view map of the significant metabolic pathways of *L. vannamei* after low-temperature storage. The *X*-axis represents pathway enrichment, and the *Y*-axis represents the pathway influence. Large sizes and dark colors represent the major pathway enrichment and high pathway impact values, respectively.

## Abbreviations

UPLC-Q-Exactive Orbitrap/MS: ultrahigh-performance liquid chromatography-Q-Exactive Orbitrap mass spectrometry; ESI^+^: positive electrospray ionization; ESI^−^: negative electrospray ionization; PC: phosphatidylcholine; PE: phosphatidylethanolamine; PI: phosphatidylinositol; PG: phosphatidylglycerol; PS: phosphatidylserine; LPC: lysophosphatidylcholine; LPE: lysophosphatidylethanolamine; LPI: lysophosphatidylinositol; LPG: lysophosphatidylglycerol; LPS: lysophosphatidylserine; Cer: ceramide; HexCer: hexosylceramide; SM: sphingomyelin; DG: diglyceride; TG: triglyceride.
