# Supplementary material for: Significantly Different Lipid Profile Analysis of Litopenaeus vannamei under Low-Temperature Storage by UPLC-Q-Exactive Orbitrap/MS
Source: Foods. 2021 Oct 29;10(11):2624. doi: 10.3390/foods10112624 (PMC8622687; doi:10.3390/foods10112624)
Supplement: Supplementary file 1 [file foods-10-02624-s001.zip › Figure S1.pdf]

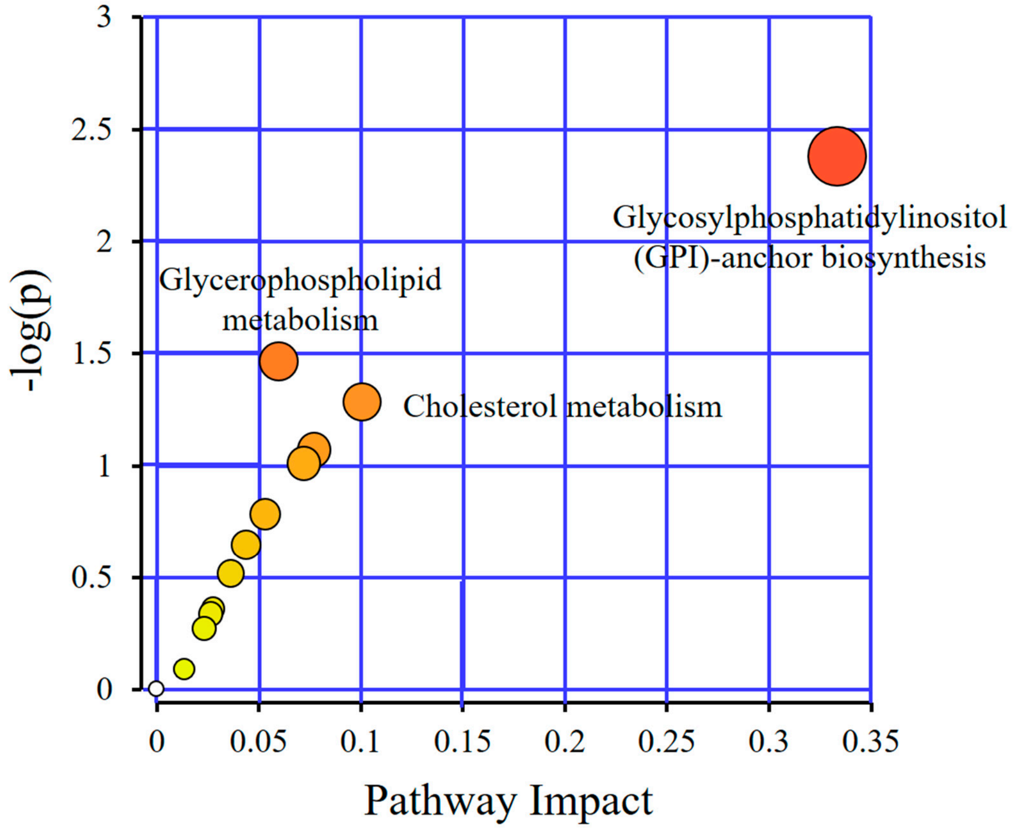

Figure S1. Metabolomic view map of the significant metabolic pathways of *L. vannamei* under low-temperatures storage. The X-axis represents pathway enrichment, and the Y-axis represents the pathway influence. Large sizes and dark colors represent the major pathway enrichment and high pathway impact values, respectively.
